# The A allele of the rs759853 single nucleotide polymorphism in the AKR1B1 gene confers risk for diabetic kidney disease in patients with type 2 diabetes from a Brazilian population

**DOI:** 10.20945/2359-3997000000432

**Published:** 2022-01-14

**Authors:** Cristine Dieter, Natália Emerim Lemos, Nathalia Rodrigues de Faria Corrêa, Felipe Mateus Pellenz, Luís Henrique Canani, Daisy Crispim, Andrea Carla Bauer

**Affiliations:** 1 Hospital de Clínicas de Porto Alegre Divisão Endócrina Porto Alegre RS Brasil Divisão Endócrina do Hospital de Clínicas de Porto Alegre, Porto Alegre, RS, Brasil; 2 Universidade Federal do Rio Grande do Sul Faculdade de Medicina Departamento de Clínica Médica Porto Alegre RS Brasil Universidade Federal do Rio Grande do Sul, Faculdade de Medicina, Departamento de Clínica Médica, Programa de Pós-graduação em Ciências Médicas: Endocrinologia, Porto Alegre, RS, Brasil; 3 Hospital de Clínicas de Porto Alegre Serviço de Nefrologia Porto Alegre RS Brasil Serviço de Nefrologia do Hospital de Clínicas de Porto Alegre, Porto Alegre, RS, Brasil

**Keywords:** *AKR1B1* gene, DNA polymorphism, diabetic kidney disease

## Abstract

**Objective:**

The *AKR1B1* gene encodes an enzyme that catalyzes the reduction of glucose into sorbitol. Chronic hyperglycemia in patients with diabetes mellitus (DM) leads to increased AKR1B1 affinity for glucose and, consequently, sorbitol accumulation. Elevated sorbitol increases oxidative stress, which is one of the main pathways related to chronic complications of diabetes, including diabetic kidney disease (DKD). Accordingly, some studies have suggested the rs759853 polymorphism in the *AKR1B1* gene is associated with DKD; however, findings are still contradictory. The aim was to investigate the association of the rs759853 polymorphism in the *AKR1B1* gene and DKD.

**Materials and methods:**

The sample comprised 695 patients with type 2 DM (T2DM) and DKD (cases) and 310 patients with T2DM of more than 10 years’ duration, but no DKD (controls). The polymorphism was genotyped by real-time PCR.

**Results:**

Allelic and genotype frequencies of this polymorphism did not differ significantly between groups. However, the A/A genotype was associated with risk for DKD after adjustment for gender, triglycerides, BMI, presence of hypertension and diabetic retinopathy, and duration of DM, under both recessive (P = 0.048) and additive (P = 0.037) inheritance models.

**Conclusion:**

Our data suggest an association between the *AKR1B1* rs759853A/A genotype and risk for DKD in Brazilians T2DM patients.

## INTRODUCTION

Diabetic kidney disease (DKD) is an important microvascular complication that affects around 40% of all patients with diabetes mellitus (DM), and is the leading cause of end-stage renal disease in individuals on renal replacement therapy. Moreover, patients with DKD have increased cardiovascular mortality compared to patients with DM without this complication (1,2). DKD is defined clinically by presence of albuminuria and/or a gradual decline in the glomerular filtration rate (GFR) (3). Known risk factors for DKD are long-lasting hyperglycemia, arterial hypertension, dyslipidemia, and genetic polymorphisms (1,4). 

Aldo-keto reductase family 1 member B (AKR1B1), also known as aldose reductase, belongs to the aldo/keto reductase superfamily and is the first enzyme of the polyol pathway, catalyzing the reduction of glucose into sorbitol using NADPH as a cofactor [reviewed in (5,6)]. This reaction is the rate-limiting step of the polyol pathway. Under chronic hyperglycemia in patients with DM, AKR1B1 affinity for glucose is high, leading to sorbitol accumulation and increased consumption of NADPH, thus reducing the available amount of this cofactor to be used in other metabolic processes, such as production of nitric oxide (5,6). Moreover, sorbitol accumulation changes cellular membrane osmotic pressure and triggers oxidative stress, long thought to be one of the main causative mechanisms of DM and its chronic complications. In the kidneys, it may trigger dysfunction and, consequently, DKD (5-7). 

In this context, some studies have demonstrated an association between single nucleotide polymorphisms (SNPs) in the *AKR1B1* gene and chronic complications of DM, including DKD (8-12). The rs759853 G/A SNP is located in the promoter region of *ARK1B1* gene and has been studied in several populations regarding a purported association with DKD. A meta-analysis performed by Cui and cols. (8) included nine case-control or cohort studies that investigated the associated between the rs759853 SNP and DKD, and showed this SNP was associated with risk for DKD in patients with type 1 DM (T1DM) or type 2 DM (T2DM) [OR = 1.52, 95% CI (1.26-1.84), P < 0.0001, for the dominant model]. However, considering that none of these studies used the current criteria for classifying kidney disease (13) and no study has been conducted in the Brazilian population, we performed a case-control study to investigate whether the *AKR1B1* rs759853 SNP is associated with DKD in patients from Southern Brazil with T2DM.

## MATERIALS AND METHODS

### Sample profile and clinical and laboratory analyses

This case-control study was conducted in accordance with the STROBE and STREGA guidelines (14,15). The sample consisted of 1,005 unrelated patients with T2DM recruited from *Hospital de Clínicas de Porto Alegre* and *Grupo Hospitalar Conceição* (Porto Alegre, Rio Grande do Sul, Brazil) between 2002 and 2013, as previously described in detail (16).

T2DM was diagnosed following American Diabetes Association guidelines (17), while DKD was diagnosed based on KDIGO guidelines (13), using urinary albumin excretion (UAE) and estimated GFR (eGFR). Patients were divided into two groups according to renal function: 1) non-DKD controls (n = 310): patients with T2DM of ≥10 years’ duration and without any degree of DKD (UAE <30 mg/g and eGFR ≥60 mL/min/1.73 m²; and 2) DKD cases (n = 695): patients with moderate (UAE 30-300 mg/g and/or eGFR 30-59 mL/min/1.73 m²) or severe DKD (UAE >300 mg/g and/or eGFR 1-29 mL/min/1.73 m²). All subjects included in the study self-declared their ethnicity as “White”.

A standard form was applied to collect data on age, age at T2DM diagnosis, T2DM duration, and drug treatment, and all subjects underwent clinical and laboratory evaluations, as reported elsewhere (18). Concisely, patients were weighed barefoot, wearing outdoor clothes, and their height was recorded. Body mass index (BMI) was calculated as weight (kg)/height (meters)^2^. Fasting serum and plasma samples were collected for laboratory measurements. Fasting glucose levels were measured using the glucose oxidase method. Glycated hemoglobin (HbA1c) quantification was performed using different methodologies; values were traceable to the Diabetes Control and Complications Trial (DCCT) (19). The Jaffé reaction was used for creatinine measurement. Total plasma cholesterol, HDL cholesterol, and triglycerides levels were measured using enzymatic methods, and UAE was quantified by immunoturbidimetry (20). EGFR was calculated using the Chronic Kidney Disease Epidemiology Collaboration (CKD-EPI) equation (21).

The protocol of this study was approved by the Research Ethics Committee of *Hospital de Clínicas de Porto Alegre* (CAAE number: 97779318700005327), and all individuals gave assent and written informed consent prior to inclusion in the study.

### Genotyping of the rs759853 SNP on the *AKR1B1* gene

DNA was extracted from blood leukocytes using a salting-out method (22). The rs759853 (G/A) SNP in the *AKR1B1* gene was genotyped by allele discrimination-real time PCR technique using a Human TaqMan SNP Genotyping Assay (Thermo Fisher Scientific, Foster City, CA, USA) specific for this SNP (assay ID = C_2795303_10). Real-time PCRs were conducted in 384-well plates (5µL total volume), using 2 ng of DNA, TaqMan ProAmp Mastermix 1× (Thermo Fisher Scientific), and TaqMan SNP Genotyping Assay 1×. Plates were then placed in the ViiA7 Real-Time PCR System (Thermo Fisher Scientific) and heated at 95 °C (10 minutes), which was followed by 50 cycles of 95 °C (15 seconds) and 62 °C (90 seconds). 

### Statistical analyses 

Allele frequencies were counted directly, and deviations from Hardy-Weinberg equilibrium (HWE) were checked using the chi-square (χ^2^) test. Genotype and allele frequencies were compared between groups using χ^2^ tests. Moreover, genotypes were compared between case and control groups considering different inheritance models, categorized accordingly to a previous study (23). Clinical and laboratory variables were compared between groups using Student’s *t* tests or χ^2^ tests, as suitable. Categorical data are shown as percentages. Normal distributions of continuous characteristics were evaluated using Kolmogorov-Smirnov and Shapiro-Wilk tests. Those variables with normal distribution are shown as mean ± SD or percentage, while those characteristics with a skewed distribution were log-transformed before analyses and are shown as median (interquartile range).

The size of association between the rs759853 genotypes and DKD was calculated using OR with 95% CI. Multivariate logistic regression analyses were used to evaluate whether the *AKR1B1* SNP was independently associated with DKD while adjusting for confounding factors. To select the confounding factors to be included in the multivariate model, we chose those variables with P < 0.250 on univariate analysis or those with a relevant biological association with DKD. Statistical analyses were done in PASW Statistics 18.0 software (SPSS, Chicago, IL), and P values < 0.05 were considered significant. 

Sample size was calculated in the OpenEpi website (www.openepi.com), using frequencies from a previous meta-analysis that evaluated the association of this SNP with DKD in Caucasian individuals with T2DM (minor allele frequency = 0.27 and OR = 1.6) (8). Therefore, the calculated sample size was 568 individuals in the case group and 256 individuals in the control group.

## RESULTS

### Sample description

The main characteristics of non-DKD patients (controls) and DKD cases are shown in Table 1. The median eGFR (mL/min per 1.73 m^2^) was 82.0 (70.0-92.0) in the non-DKD group and 46.0 (20.0-63.0) in patients with DKD (P < 0.0001), while the median UAE (mg/g) was 5.1 (3.0-10.9) in controls and 77.7 (23.9-349.3) in the DKD group (P < 0.0001). As expected, arterial hypertension and diabetic retinopathy (DR) were more prevalent in the DKD group (P < 0.0001). Also, HDL cholesterol was lower and triglyceride levels were higher in DKD patients compared to non-DKD patients (P < 0.0001 for both). Males comprised 53.7% of the cases and 36.2% of the control group (P < 0.0001).

**Table 1:** Clinical and laboratory characteristics of non-DKD patients (controls) and DKD cases

Characteristic	Non-DKD patients (n = 310)	DKD cases (n = 695)	P *
Age (years)	67.4 ± 10.5	66.9 ± 11.3	0.530
Gender (% male)	36.2	53.7	<0.0001
BMI (kg/m²)	28.8 ± 5.2	28.7 ± 5.1	0.970
HbA1c (%)	7.4 ± 1.6	7.6 ± 2.0	0.126
Hypertension (%)	81.2	90.0	<0.0001
Age at DM diagnosis (years)	47.0 ± 10.4	47.8 ± 11.1	0.282
T2DM duration (years)	20.3 ± 8.3	18.9 ± 10.6	0.024
Total cholesterol (mg/dL)	194.6 ± 47.0	194.6 ± 51.0	>0.999
Triglycerides (mg/dL)	133.5 (96.2-186.0)	158.5 (111.0-240.7)	<0.0001
HDL cholesterol (mg/dL)	47.4 ± 12.4	42.9 ± 11.9	<0.0001
LDL cholesterol (mg/dL)	114.2 ± 42.9	114.1 ± 45.3	0.994
Creatinine (µg/dL)	0.8 (0.7 -0.9)	1.3 (1.0-2.8)	<0.0001
eGFR (mL/min per 1.73 m2)	82.0 (70.0-92.0)	46.0 (20.0-63.0)	-
UAE (mg/g)	5.1 (3.0-10.9)	77.7 (23.9-349.3)	-
DR (%)	43.6	61.8	<0.0001

Variables are shown as mean ± SD, median (25th-75th percentiles) or %.

* P values were computed using Student’s *t* tests or χ^2^ tests, as appropriate. BMI: body mass index; DKD: diabetic kidney disease; DR: diabetic retinopathy; eGFR: estimated glomerular filtration rate; HbA1c: glycated hemoglobin; T2DM: type 2 diabetes mellitus; UAE: urinary albumin excretion.

### Genotype and allele frequencies in DKD patients and controls

Genotype and allele frequencies of the rs759853 (G/A) SNP in the *AKR1B1* gene in cases with DKD and controls without this complication are described in Table 2. Genotype frequencies of this SNP were consistent with the HWE in the control group (P = 0.817) and did not differ significantly between cases and controls (P = 0.410). Additionally, the minor allele frequency (A) of the rs759853 SNP did not differ between groups (39% in cases *vs.* 37% in controls, P = 0.327). However, on multivariate analysis, the A/A genotype was significantly associated with risk for DKD [OR = 1.630, 95% CI (1.025-2.618), P = 0.039], adjusting for gender, triglycerides, BMI, presence of hypertension, DR, and duration of DM (Table 2). Accordingly, the A/A genotype remained associated with risk for DKD under both recessive [OR 1.548, 95% CI (1.004-2.388), P = 0.048] and additive [OR = 1.659, 95% CI (1.030-2.672), P = 0.037] inheritance models, adjusting for the same above-mentioned variables. 

**Table 2:** Genotype and allele frequencies of *AKR1B1* rs759853 SNP in non-DKD (controls) and DKD cases

	Non-DKD patients (n = 310)	DKD cases (n = 695)	P *	Adjusted OR (95% CI) / P†
Genotype				
G/G	127 (41.0)	275 (39.6)	0.410	1
G/A	137 (44.2)	293 (42.2)		1.115 (0.786-1.583)/ 0.541
A/A	46 (14.8)	127 (18.2)		1.630 (1.025-2.618)/ 0.039
Allele				
G	0.63	0.61	0.327	-
A	0.37	0.39		
**Recessive model**				
G/G + G/A	264 (85.2)	568 (81.7)	0.214	1
A/A	46 (14.8)	127 (18.3)		1.548 (1.004-2.388)/ 0.048
**Additive model**				
G/G	127 (73.4)	275 (68.4)	0.271	1
A/A	46 (26.6)	127 (31.6)		1.659 (1.030-2.672)/ 0.037
**Dominant model**				
G/G	127 (41.0)	275 (39.6)	0.727	1
G/A + A/A	183 (59.0)	420 (60.4)		1.247 (0.901-1.726)/ 0.184

Data are shown as number (%) or proportion.

* P values were calculated using χ^2^ tests (except P values for genotype comparison between groups, which were obtained by univariate logistic regression analysis).

† P values and OR (95% CI) obtained using logistic regression analyses adjusting for gender, triglycerides, BMI, presence of hypertension and DR, and duration of T2DM.

Exploratory analyses were performed to compare clinical and laboratory characteristics between T2DM patients stratified by presence of the rs759853 A/A genotype under the recessive model (Table 3). Mean age, HbA1c, age at diagnosis, duration of DM, total cholesterol, triglycerides, HDL, LDL, and UAE did not differ between patients carrying the A/A genotype and those with the G/G + G/A genotype (P > 0.050). Additionally, frequencies of male sex, hypertension, and DR did not differ between groups (P > 0.050). 

**Table 3:** Clinical and laboratory characteristics of T2DM patients stratified by presence of the A/A genotype of the *AKR1B1* rs759853 SNP (recessive model)

Characteristic	G/G + G/A (n = 832)	A/A (n = 173)	P *
Age (years)	67.1 ± 11.0	67.1 ± 11.3	0.931
Gender (% male)	47.8	50.9	0.511
BMI (kg/m²)	28.6 ± 5.0	29.3 ± 5.5	0.162
HbA1c (%)	7.6 ± 1.9	7.5 ± 2.0	0.523
Hypertension (%)	87.4	87.0	0.969
Age at T2DM diagnosis (years)	47.5 ± 10.7	47.8 ± 11.8	0.705
DM duration (years)	19.4 ± 9.9	19.1 ± 10.4	0.681
Total cholesterol (mg/dL)	194.5 ± 49.7	195.2 ± 50.2	0.882
Triglycerides (mg/dL)	149.0 (105.0-221.2)	151.5 (109.7-225.2)	0.858
HDL cholesterol (mg/dL)	44.4 ± 12.3	44.0 ± 12.0	0.663
LDL cholesterol (mg/dL)	114.4 ± 44.8	112.8 ± 43.2	0.690
Creatinine (µg/dL)	1.1 (0.8-1.8)	1.0 (0.8-1.5)	0.417
eGFR (mL/min per 1.73 m^2^)	60.0 (32.0-82.0)	60.0 (40.5-82.5)	0.246
UAE (mg/g)	22.0 (5.0-142.0)	32.8 (5.5-143.5)	0.482
DR (%)	57.0	51.2	0.213

Variables are shown as mean ± SD, median (25th-75th percentiles) or %.

* P value was computed using Student’s *t* tests or χ^2^ tests, as appropriate. BMI: body mass index; DKD: diabetic kidney disease; DR: diabetic retinopathy; eGFR: estimated glomerular filtration rate; HbA1c: glycated hemoglobin; T2DM: type 2 diabetes mellitus; UAE: urinary albumin excretion.

## DISCUSSION

AKR1B1 acts on the polyol pathway by catalyzing the reduction of glucose to sorbitol. Under hyperglycemic environments, this pathway leads to intracellular buildup of sorbitol, causing tissue damage – as observed in the microvascular complications of diabetes, including DKD (24). Hence, *AKR1B1* polymorphisms have been associated with DM and its complications. The rs759853 SNP in the promoter region of the *AKR1B1* gene has been the most studied SNP in this gene regarding DKD (8). Therefore, we sought to analyze the association between the rs759853 SNP and susceptibility to DKD in a Southern Brazilian population. Our results show the A/A genotype was associated with risk for DKD. This is the first study to replicate the association between the rs759853 SNP and DKD in a Latin American population, and using both UAE and eGFR measurements for DKD classification. 

In agreement with our results, other studies have demonstrated the association of rs759853 SNP with risk for DKD in different populations (9-12,25-27). Neamat-Allah and cols. (9) reported the association of the G/A + A/A genotypes with risk for DKD in T1DM and T2DM patients from England and Ireland. In the same line, in American Caucasians with T1DM, the A/A genotype was reported as a risk factor for DKD, with the A allele frequency being higher in those with DKD compared to those without this complication (41.2% *vs.* 32.9%, P = 0.014) (10). The A/A genotype frequency of this SNP was also higher in Japanese T2DM patients with DKD compared to normoalbuminuric controls, and was associated with risk for DKD after adjustment for covariables (OR = 4.3; 95% CI 1.1-6.0) (25). In a prospective cohort comprising 1,074 Chinese T2DM patients, those who developed cardiorenal complications over 8 years of follow-up had a higher frequency of the G/A + A/A genotypes (44% *vs.* 35%, P = 0.008) and A allele (27% *vs.* 22%, P = 0.026) in comparison to those who did not develop any complication (12). In contrast, no association was found between this SNP and DKD in another sample of Chinese T2DM patients (26). Cui and cols. (8) performed a meta-analysis of nine case-control or cohort studies (totaling 4,735 T1DM and T2DM patients) that investigated the association between the rs759853 SNP and DKD, and showed significant associations between this SNP and susceptibility to DKD in both T1DM and T2DM groups, under different inheritance models. Moreover, this association remained in T2DM patients stratified by ethnicity (Caucasians and Asian patients). On the other hand, no association was observed between this SNP and progression of DKD (8). 

The rs759853 SNP in *AKR1B1* has also been investigated regarding its association with DR; however, findings are still controversial (28,29). Kaur and Vanita (28) analyzed 926 North Indian T2DM patients and reported an association between the A/A genotype and risk for DR (OR = 1.61, 95% CI 1.39-2.28). In contrast, a study comprising 268 Chinese T2DM patients found no significant difference in rs759853 genotypes between patients with and without DR (P = 0.400) (29). Furthermore, Cao and cols. (30) showed in a meta-analysis of 21 publications that rs759853 was not associated with DR. Interestingly, after subgroup analysis by DM type, this SNP conferred protection against DR onset in patients with T1DM (additive model: OR = 0.33, 95% CI 0.17-0.67; dominant model: OR = 0.49, 95% CI 0.36-0.68; recessive model: OR = 0.48, 95% CI 0.28-0.83) (30). Of note, in the present study, DR was included as a covariate in the logistic regression analyses. 

*AKR1B1* gene and protein expressions have also been studied in diabetic patients (31,32). Hodgkinson and cols. (33) cultured peripheral blood mononuclear cells from DM patients with high glucose for 5 days and showed that *AKRKB1* mRNA levels were higher in those cells collected from DKD patients compared to non-DKD patients and healthy subjects. Lewko and cols. (34) reported that *AKR1B1* gene and protein expressions were elevated in mouse podocytes cultured with high glucose compared to cells cultured under normal glucose concentration. In kidneys from patients with and without DM, AKR1B1 activity was higher in glomeruli and small arteries of those patients with DKD compared to the non-DKD group (35). Interestingly, a recent study found hypomethylation of the *AKR1B1* gene in T2DM DKD cases compared to non-DKD patients (36). Moreover, *AKR1B1* methylation levels were negatively correlated with UAE levels in DKD patients (36). 

Some aspects may have influenced the findings of the present study. First, we cannot exclude a population stratification bias when investigating our samples, as only White individuals enrolled in the study. Second, we cannot rule out the occurrence of type II error during the statistical analyses. Even though we had more than 80% power (α = 0.05) to detect an OR ≥ 1.6 for DKD risk, we cannot rule out the possibility that the *AKR1B1* rs759853A allele could be associated with DKD at lower ORs. Third, we found an association between this SNP and risk for DKD only after adjusting for covariates; however, this analysis is extremely important, since DKD is a multifactorial disease, caused either by activation of glucose-dependent pathways as well as by the presence of hypertension and obesity in patients with T2DM.

In conclusion, our study indicates that the A/A genotype of rs759853 SNP in the *AKR1B1* gene is a risk factor for DKD in a Southern Brazilian population. This association has also been demonstrated in other populations of different ethnic origins and is biologically plausible, considering the involvement of AKR1B1 in the polyol pathway and its relation with DM and its complications. 
